# Supplementary material for: Implementation of vascular surgery teleconsultation during the COVID-19 pandemic: Insights from the outpatient vascular clinics in a tertiary care hospital in Qatar
Source: PLoS One. 2021 Sep 30;16(9):e0257458. doi: 10.1371/journal.pone.0257458 (PMC8483384; doi:10.1371/journal.pone.0257458)
Supplement: Attachment — Submitted filename: Plos one.docx [file pone.0257458.s001.docx]

In the present manuscript authors have analyzed the frailty and benefit of telecommunication as tool for organizing vascular surgery outpatient clinic during Covid-19 era. This is a timely topic for the research and authors have a large cohort. Authors have a valid scope for the study “The goal of this study is to describe the utilization of telemedicine services in the vascular surgery clinic in a tertiary hospital in Qatar and explore some considerations for practice from the Qatar context that may be relevant in other settings globally” The major criticism is the grouping of this nice amount of valuable data, which can provide potentially interesting valuable new data on which patient sub-groups can be managed with telecommunication and who really require face-to-face contact to out-patient clinic. Comparing groups like nationals versus non-nationals does not provide globally relevant new data. The over 10 000 visits would provide more general importance if divided based on clinical presentation of vascular disease or requirement of vascular services (access patients etc). Comparing numbers of venous patients C1-3 versus C4-6 telecommunication versus face-to-face, similarly numbers treated and referred before and after Covid-19 pandemic, claudication versus CLI patients, AAA, internal consultations (nephrology, neurology) etc would provide more general new insight to utility of telemedicine in special patient groups and workload of vascular services during pandemic. This data should be available on authors data base?

Abstract:

Background. Last sentence not fluent and could be pasted to another paragraph?

Objectives and methods should be more accurate.

Results is hard to follow, and it is impossible to know where authors present data and numbers from outpatient clinic and when numbers present the workload of vascular clinic. This is also major criticism for all sections of the manuscript.

Conclusions first sentence is on focus and as stated in the major criticism the present study has potential answering many important aspects of vascular out-patients clinic.

Introduction is nice

Methods: please see above. The grouping of the data could be more relevant. Table 1 figures 1-2 could be more readable? Statistical analyses especially table 2 should be presented more clearly in methods section and in the legend.

Results table 2 is hard to read (p value chi test between which values?) more accurate presentation and legend would be helpful and provide sufficient information on what is presented. Figure 3 why not present year 2020 together with figure 5 on same panel? This would be more informative? It is not clear if authors present number of out-patient clinic or vascular department. Maybe mixed? State accurately what numbers stand for.

Discussion would be nice to begin with the major observations of the study. Second and third paragraph essential for the manuscript? If authors could demonstrate significant differences between urgent and elective vascular patient workload during pandemics and possibly these patient groups vary in requirements of out-patient contact (face-to-face or telecommunication)? Also, the multidisciplinary patient groups are interesting on this concept.
