# Supplementary material for: Implementation of vascular surgery teleconsultation during the COVID-19 pandemic: Insights from the outpatient vascular clinics in a tertiary care hospital in Qatar
Source: PLoS One. 2021 Sep 30;16(9):e0257458. doi: 10.1371/journal.pone.0257458 (PMC8483384; doi:10.1371/journal.pone.0257458)
Supplement: Attachment — Submitted filename: reply3.doc [file pone.0257458.s004.doc]

**Thanks for the reviewers and editor. There was only one comment that already has been addressed.**

**Comments to the Author**

1. If the authors have adequately addressed your comments raised in a previous round of review and you feel that this manuscript is now acceptable for publication, you may indicate that here to bypass the “Comments to the Author” section, enter your conflict of interest statement in the “Confidential to Editor” section, and submit your "Accept" recommendation.

Reviewer #2: All comments have been addressed

Reviewer #3: All comments have been addressed

Reviewer #4: All comments have been addressed

2. Is the manuscript technically sound, and do the data support the conclusions?

The manuscript must describe a technically sound piece of scientific research with data that supports the conclusions. Experiments must have been conducted rigorously, with appropriate controls, replication, and sample sizes. The conclusions must be drawn appropriately based on the data presented.

Reviewer #2: Yes

Reviewer #3: Yes

Reviewer #4: Yes

3. Has the statistical analysis been performed appropriately and rigorously?

Reviewer #2: Yes

Reviewer #3: N/A

Reviewer #4: Yes

4. Have the authors made all data underlying the findings in their manuscript fully available?

The [PLOS Data policy](http://www.plosone.org/static/policies.action#sharing) requires authors to make all data underlying the findings described in their manuscript fully available without restriction, with rare exception (please refer to the Data Availability Statement in the manuscript PDF file). The data should be provided as part of the manuscript or its supporting information, or deposited to a public repository. For example, in addition to summary statistics, the data points behind means, medians and variance measures should be available. If there are restrictions on publicly sharing data—e.g. participant privacy or use of data from a third party—those must be specified.

Reviewer #2: Yes

Reviewer #3: Yes

Reviewer #4: Yes

5. Is the manuscript presented in an intelligible fashion and written in standard English?

PLOS ONE does not copyedit accepted manuscripts, so the language in submitted articles must be clear, correct, and unambiguous. Any typographical or grammatical errors should be corrected at revision, so please note any specific errors here.

Reviewer #2: Yes

Reviewer #3: Yes

Reviewer #4: Yes

6. Review Comments to the Author

Please use the space provided to explain your answers to the questions above. You may also include additional comments for the author, including concerns about dual publication, research ethics, or publication ethics. (Please upload your review as an attachment if it exceeds 20,000 characters)

Reviewer #2: All presented comments are addressed.

Reviewer #3: It is clear that a lot of hard work has gone in to preparing this manuscript, however it is my feeling that rather than generating new knowledge this is more of an audit/local service evaluation and perhaps could be usefully relayed in a short letter to the editor rather than a full manuscript.

**Reply: Thanks, This is the first paper of this kind in the Middle East (and few worldwide) that addresses the importance and feasibility of teleconsultation in the field of vascular surgery during the unprecedented pandemic, therefore full manuscript is needed**

Reviewer #4: Dear author,

Your submission is very well-written and was able to adress all suggested comments raised from the previous revision.
There is still a minor mistake at page 22: "Vascular surgery for arthrosclerosis" instead of atherosclerosis, which should be corrected.

**Reply: thanks, corrected**

7. PLOS authors have the option to publish the peer review history of their article ([what does this mean?](https://journals.plos.org/plosone/s/editorial-and-peer-review-process#loc-peer-review-history)). If published, this will include your full peer review and any attached files.


If you choose “no”, your identity will remain anonymous but your review may still be made public.


**Do you want your identity to be public for this peer review?** For information about this choice, including consent withdrawal, please see our [Privacy Policy](https://www.plos.org/privacy-policy).

Reviewer #2: No

Reviewer #3: No

Reviewer #4: No
